# The Influence of Lignin Diversity on the Structural and Thermal Properties of Polymeric Microspheres Derived from Lignin, Styrene, and/or Divinylbenzene

**DOI:** 10.3390/ma12182847

**Published:** 2019-09-04

**Authors:** Marta Goliszek, Beata Podkościelna, Olena Sevastyanova, Barbara Gawdzik, Artur Chabros

**Affiliations:** 1 Department of Polymer Chemistry, Faculty of Chemistry, Maria Curie-Sklodowska University, M. Curie-Sklodowska Sq. 3, 20-031 Lublin, Poland; 2 Department of Fibre and Polymer Technology, KTH Royal Institute of Technology, Teknikringen 56-58, SE-10044 Stockholm, Sweden; 3 KTH Royal Institute of Technology, Wallenberg Wood Science Center, Teknikringen 56-58, SE-10044 Stockholm, Sweden

**Keywords:** lignin, microspheres, composites, polymeric material, fractionation, porosity

## Abstract

This work investigates the impact of lignin origin and structural characteristics, such as molecular weight and functionality, on the properties of corresponding porous biopolymeric microspheres obtained through suspension-emulsion polymerization of lignin with styrene (St) and/or divinylbenzene (DVB). Two types of kraft lignin, which are softwood (*Picea abies* L.) and hardwood (*Eucalyptus grandis*), fractionated by common industrial solvents, and related methacrylates, were used in the synthesis. The presence of the appropriate functional groups in the lignins and in the corresponding microspheres were investigated by attenuated total reflectance Fourier transform infrared spectroscopy (ATR/FT-IR), while the thermal properties were studied by differential scanning calorimetry (DSC). The texture of the microspheres was characterized using low-temperature nitrogen adsorption. The swelling studies were performed in typical organic solvents and distilled water. The shapes of the microspheres were confirmed with an optical microscope. The introduction of lignin into a St and/or DVB polymeric system made it possible to obtain highly porous functionalized microspheres that increase their sorption potential. Lignin methacrylates created a polymer network with St and DVB, whereas the unmodified lignin acted mainly as an eco-friendly filler in the pores of St-DVB or DVB microspheres. The incorporation of biopolymer into the microspheres could be a promising alternative to a modification of synthetic materials and a better utilization of lignin.

## 1. Introduction

Interest in producing bio-polymer-based materials from renewable resources has increased recently as a step toward sustainable development, by utilizing technologies that are safe for the environment [1,2,3,4,5]. In response to the increasing environmental concern and diminishing resources of petrochemicals, the use of polymers from renewable resources is a promising alternative [6,7].

Lignocellulosic biomass contains one of the most abundant renewable forms of carbon and is, thus, regarded as a logical feedstock to replace traditional fossil resources [8,9]. Biopolymers derived from lignocellulosic biomass, agricultural crops, or wood are currently considered to be the main resource for developing biodegradable and renewable polymeric materials [10,11]. Lignin as a phenolic component of biomass is the most abundant natural substance composed of aromatic moieties and the second most abundant naturally occurring terrestrial polymer after cellulose [12,13]. It is a linker between cellulose bundles, giving rigidity and strength to the cell walls and resistance toward an attack by microorganisms, which provides a route for the transport of water in plant stems. Structurally, lignin is an amorphous macromolecule formed from three phenylpropanoid units: p-coumaryl alcohol, sinapyl alcohol, and coniferyl alcohol connected by ether and carbon-carbon bonds [14], while the functional groups present in lignin (namely hydroxyl, methoxyl, carbonyl, and carboxylic groups) [15] make it possible to be functionalized through various chemical modifications [16,17,18,19,20]. Large quantities of lignin are released by the kraft process during the production of pulp and paper from wood. Lignin has been mainly regarded as a cheap fuel for the process, but recent concerns for more efficient utilization of natural resources have drawn attention to its further valorization [14,21,22,23,24,25,26,27,28,29]. However, more effort is needed for the engineering of lignin-based value-added materials to make them financially competitive.

To overcome the above-mentioned limitations of using natural polymers, they can be used in composites and blends with synthetic polymers, which is an effective way to obtain a desirable combination of properties that are absent in the individual components [30,31]. In the last few decades, lignin has been added to various polymers and the materials obtained have sometimes been called blends, and, in other cases, composites, but it is not always clear whether lignin forms a blend or acts as a filler in the composite material [32]. For example, Yin et al. [33] prepared a cross-linked biomass-polymer composite by mixing lignin with an epoxy resin and polyamine using a hot press molding process. They showed that the epoxy resin could be cured by lignin, and that a good interfacial combination was formed between the components. Jesionowski et al. [34] obtained advanced multifunctional silica/lignin composite materials. The composite powders obtained were blended with multiwalled carbon nanotubes. The electrochemical activity assessed by cyclic voltammetry revealed the presence of a redox system assigned to a lignin-derived quinone/hydroquinone couple. He et al. [35] prepared a polyaniline-lignin composite and demonstrated its good adsorption of silver ions, which indicates that the lignin unit could play a vital role in the chelation of silver ions, while the polyaniline unit was important for electrical conduction. Ballner et al. [36] obtained lignocellulose nanofiber-reinforced polystyrene from composite microspheres produced by suspension polymerization. They reported superior impact toughness and improved bending strength for the composite in comparison with unreinforced polystyrene.

One of the main issues in the commercial production of value-added lignin products is the heterogeneous nature of lignin, which makes it difficult to standardize the qualities and properties of the products from lignin. Increased attention has recently been given to the fractionation into more homogeneous preparations using organic solvents, ionic liquids, or ultrafiltration [37,38,39,40,41,42,43,44,45]. For example, Li et al. [38] investigated sequential solvent fractionation of heterogeneous bamboo organosolv lignin. The starting lignin and obtained fractions were compared in terms of functional groups and molecular weight distribution. It was concluded that sequential solvent fractionation can be a useful method to prepare homogeneous lignin with desirable functional groups for further processing. Jääskeläinen et al. [40] have reported a precipitation fractionation method for kraft lignin based on aqueous organic solvents. In this approach, moisture containing lignin can be subjected to fractionation directly without initial drying, which is a great advantage for large-scale applications. It was shown that the molar mass and functionalities of the precipitated lignin fractions depended on the ratio between solvent and water in the precipitation step. Tagami et al. [41] compared the impact of sequential solvent fractionation on the molecular weight, composition, and contents of functional groups of industrial softwood and hardwood kraft lignins. In this work, it was demonstrated that the antioxidant activity, chemical structure, heating values, and thermal and adsorption properties of lignin can be tuned by such processing. The results obtained provide useful information for targeted uses of lignin raw material. Toledano et al. [43] investigated ultrafiltration and selective precipitation as two different methods for lignin fractionation and confirmed that both fractionation processes influence the properties of the obtained lignin. Lauberts et al. [45] investigated fractionation of technical lignin using ionic liquids for further application as antioxidants. It was shown that fractionated lignins with improved polydispersity resulted in higher antioxidant activity as compared to non-fractionated lignins. Sevastyanova et al. [46] produced a wide range of lignin fractions by ultrafiltration of black liquor using ceramic membranes with a different Mw cut-off. Investigation of the thermal properties of such fractions revealed the possibility to tailor certain parameters by choice of membrane. Lignin fractions with well-defined characteristics could be a predictive tool for the development of high-performance lignin-based systems [47,48,49].

Polymer porous microspheres obtained by a suspension-polymerization method have great potential in the sorption processes [17]. The addition of lignin into the polymeric system results in more eco-friendly materials [31,50]. In our previous work [50] on the synthesis of microspheres, we used a commercial kraft lignin and low molecular weight kraft lignin obtained by ultrafiltration of industrial black liquor using a ceramic membrane with Mw cut-off of 5 kD. The specific surface area of these materials decreased with increasing lignin content. The main aim of this work is to investigate the impact of lignin fractionation on the properties of the biopolymeric microspheres obtained.

## 2. Materials and Methods

### 2.1. Chemicals and Materials

Softwood (*Picea abies* L.) and hardwood (*Eucalyptus grandis*) lignins were obtained by the kraft process and purified according to the LignoBoost technology protocol [51]. The moisture content was 6.4% and 5.1%, respectively, and ash content was 0.6% and 1.2%, respectively. Methacryloyl chloride, styrene, bis(2-ethylhexyl)sulfosuccinate sodium salt, methylene chloride, trimethylamine, magnesium sulfate, and benzyl alcohol were purchased from Sigma-Aldrich. α,α’-bis-isobutyronitrile (AIBN) and divinylbenzene (DVB) were obtained from Merck (Darmstadt, Germany) (62.2% of 1,4-divinylbenzene, 0.2% of 1,2-divinylbenzene, and ethylvinylbenzene were washed with 3% aqueous sodium hydroxide solution before use). Ethyl acetate (reagent grade) and ethanol (absolute) for the lignin fractionation were purchased from Sigma-Aldrich (Stockholm, Sweden), while methanol (analytical grade) was purchased from VWR (Stockholm, Sweden). Acetone, methanol, tetrahydrofuran (THF), chloroform, toluene, decan-1-ol, and acetonitrile for swelling studies were purchased from Avantor Performance Materials (Gliwice, Poland).

### 2.2. Lignin Fractionation, Modification, and Characterization

Lignins were fractionated with ethyl acetate, ethanol, or methanol by a two-step solvent fractionation, as described in Reference [52]. Additionally, 20 g of lignin (95% dryness) was added to 200 mL of corresponding solvent and stirred for 2 h at room temperature. After that, the insoluble fraction was separated with filter paper, dried overnight in a vacuum oven VACUCELL, MMM Medcenter EINRICHTUNGEN GmbH (Planegg, Germany) at 40 °C, and subjected to the treatment with the next solvent. Hardwood lignin was fractionated by ethyl acetate followed by ethanol, while softwood lignin extraction with ethyl acetate was followed by methanol. The lignin fraction dissolved in the corresponding solvent was recovered by concentrating the solution on a rotary evaporator IKA TV10 basic, VWR (Stockholm, Sweden) substituting the solvent with water by subsequent freeze-drying of the aqueous suspension.

The molecular weight characteristics of lignin samples were carried out using a size exclusion chromatography (SEC) using a Waters instrument consisting of a 515 HPLC pump, 2707 autosampler, and 2998 photodiode array detector (Waters Sverige AB, Sollentuna, Sweden).

The contents of functional groups were calculated by the ^31^P NMR method using Bruker Avance 400 MHz spectrometer, as described in References [41,52].

Lignin fractions were modified with methacryloyl chloride, as described in Reference [53]. The lignin sample and methylene chloride were placed with triethylamine in an ice bath and stirred. Then, methacryloyl chloride was added dropwise and a reaction proceeded for 1 h at 5 °C and for an additional hour at room temperature. The obtained material was filtered off, washed three times with water to remove trimethylamine hydrochloride, extracted with methylene chloride, and purified using a chromatographic column.

The successful chemical modification was confirmed using the Fourier transform spectroscopy with attenuated total reflection (ATR/FT-IR). ATR/FT-IR spectra were recorded using a Bruker TENSOR 27 spectrometer containing a diamond crystal (Ettlingen, Germany). The spectra were recorded in the range of 600–4000 cm^−1^ with 32 scans per spectrum at a resolution of 4 cm^−1^.

### 2.3. Synthesis of Microspheres

Styrene (St) and/or divinylbenzene (DVB) were copolymerized in an aqueous medium with different solvent fractions of lignin: Spruce-ethyl acetate (SL-ea), Spruce-methanol (SL-m), Eucalyptus-ethyl acetate (EL-ea), and Eucalyptus-ethanol (EL-e) using a suspension-emulsion polymerization method, which has previously been described in detail [50]. Unmodified lignin (L) and lignin modified with methacryloyl chloride (L-Met) were used in the synthesis of microspheres. Experimental parameters are presented in Table 1. A constant amount of benzyl alcohol as pore-forming diluent (14 mL) and DVB as a crosslinking agent (5 g) were used in every synthesis.

### 2.4. Characterization Methods of Microspheres

The ATR/FT-IR spectra were obtained as described in Section 2.2.

The calorimetric measurements were carried out in a Netzsch DSC 204 calorimeter (Selb, Germany) operated in a dynamic mode. The dynamic scans were performed at a heating rate of 10 °C min^−1^, the first scan being from 20 °C to a maximum of 110 °C to remove any adsorbed moisture, and the second from 25 °C to 550 °C in a nitrogen atmosphere (30 cm^3^ min^−1^). The mass of the sample was about 5–10 mg. An empty aluminum crucible was used as a reference.

Characterization of the porous structure was performed using Micrometrics Inc., ASAP 2405 adsorption analyzer (Norcross, GA, USA). Before the analysis, all the materials were degassed at 120 °C. The specific surface area was calculated according to the Brunauer-Emmett-Teller (BET) method, by assuming that the area of a single nitrogen molecule is 16.2 Å^2^. The pore volumes and pore size distributions were determined by the Barrett-Joyner-Halenda (BJH) method.

The swelling coefficients (B) were determined from the equilibrium swelling in chosen organic solvents and distilled water, which are calculated using the equation below.

(1) $$B=\frac{V_{s}-V_{d}}{V_{d}}\cdot 100\%$$

where V_s_ is the volume after swelling and V_d_ is the volume of the dry sample.

The appearances and morphologies of the microspheres were studied using a Malvern, MORPHOLOGI G3 optical microscope (Malvern, UK).

## 3. Results and Discussion

By using a fractionation protocol as described in Reference [52], the kraft lignin fractions of different molecular weight but with very similar content of functional groups were produced for hardwood and softwood lignins (SL-ea vs. EL-ea and SL-m vs. EL-e) (Table 2). Generally, for each type of lignin, the fractions with higher molecular weight, SL-m and EL-e, had a higher content of aliphatic -OH groups. Hardwood lignin fractions, EL-ea and EL-e, had half as many condensed guaiacyl Ph-OH groups as corresponding softwood lignin fractions, SL-ea and SL-m, which is in accordance with previously reported results [29]. Gordobil et al. [29] concluded that this structural feature most likely affects the chemical reactivity of lignin as a higher content of vinyl groups per lignin phenylpropanoid unit was introduced on methacrylation for lignin samples that have a lower degree of condensation.

**Figure 1:**
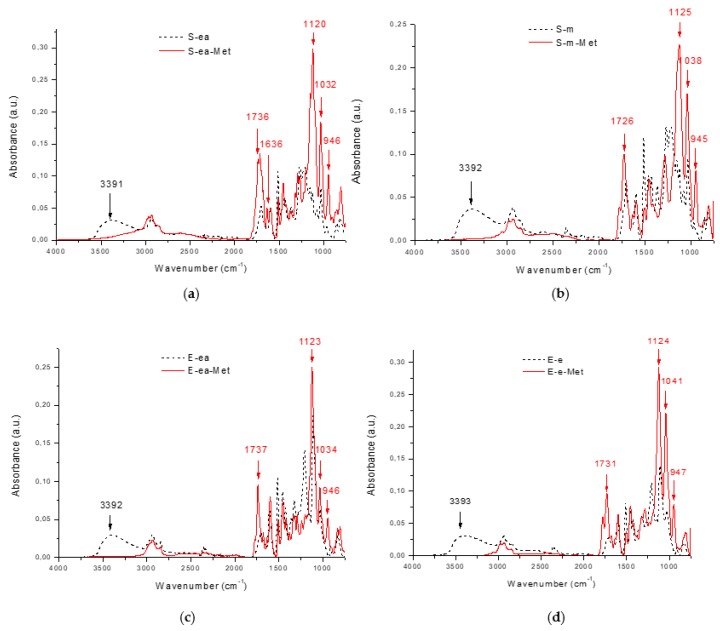
ATR/FTIR spectra of lignins: (**a**) SL-ea, (**b**) SL-m, (**c**) EL-ea, and (**d**) EL-e, before and after modification with methacryloyl chloride.

Four solvent fractions of lignin were successfully modified with methacryloyl chloride, as shown in their ATR/FT-IR spectra (Figure 1a–d). After modification, a reduction in the signal intensity of hydroxyl groups at 3391–3393 cm^−1^ [54] was observed, and new strong signals from stretching vibrations of carbonyl groups at 1726–1737 cm^−1^ were clearly visible. The new signal at 1636 cm^−1^ corresponding to C=C bonds [29] indicates the presence of methacrylate units in the lignin molecule, particularly in the SL-ea-Met lignin. The signals at 1120–1125 cm^−1^ and 1032–1041 cm^−1^ are attributed to C-O and C-O-C stretching vibrations in acrylates. The bands at 946 cm^−1^ can be attributed to a terminal C=CH_2_ bending vibration from the methacrylate groups in the lignin samples [55].

**Figure 2:**
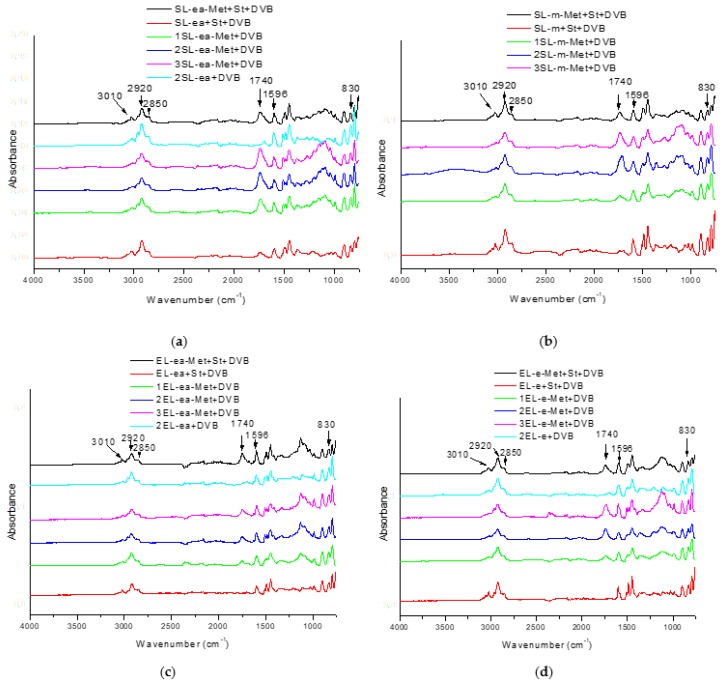
ATR/FTIR spectra of polymers with (**a**) SL-ea, (**b**) SL-m, (**c**) EL-ea, and (**d**) EL-e.

ATR/FT-IR spectra of the materials are presented in Figure 2a–d. The spectroscopic evaluation proved that the synthesis of lignin-containing microspheres had been successful, manifested by the presence of characteristic bands of appropriate functional groups. The increasing lignin content reflected in the increasing intensity of these bands agreed entirely with the expectations. In the spectra of the materials containing styrene, a signal was visible at 3010 cm^−1^, which can be attributed to stretching and deformation vibrations of C-H and confirm that styrene has been incorporated into the structure of the microspheres. Bands at 2920 and 2850 cm^−1^ can be assigned to the asymmetric and symmetric stretching vibrations of C-H in -CH_2_- groups [56] present in lignin, as well as in DVB and/or St. For the materials with St, signals between 3000–2780 cm^−1^ assigned to C(sp^3^)-H bonds are more intense due to the formation of a polymer backbone [57]. Signals at 1740 cm^−1^ attributed to stretching vibrations of carbonyl groups were observed for the materials containing modified lignin. The absorption peaks at 1596 and 1510 cm^−1^ can be assigned to C = C of aromatic skeletal vibrations from lignin, DVB, and/or St. The slight increase in intensity of these signals with increased lignin content indicate the incorporation of aromatic moieties from lignin into the synthesized materials. An additional increase in intensity of these signals indicate the presence of St in the polymer system. The signals ranging from 1200 to 1000 cm^−1^ are due to C-O-C stretching vibrations in acrylates. Their intensity increased with increasing content of modified lignin in the materials. The bands visible at around 830 cm^−1^ on all the presented spectra are attributed to aromatic C-H out-of-plane deformation vibrations, which are present in all the components used.

**Figure 3:**
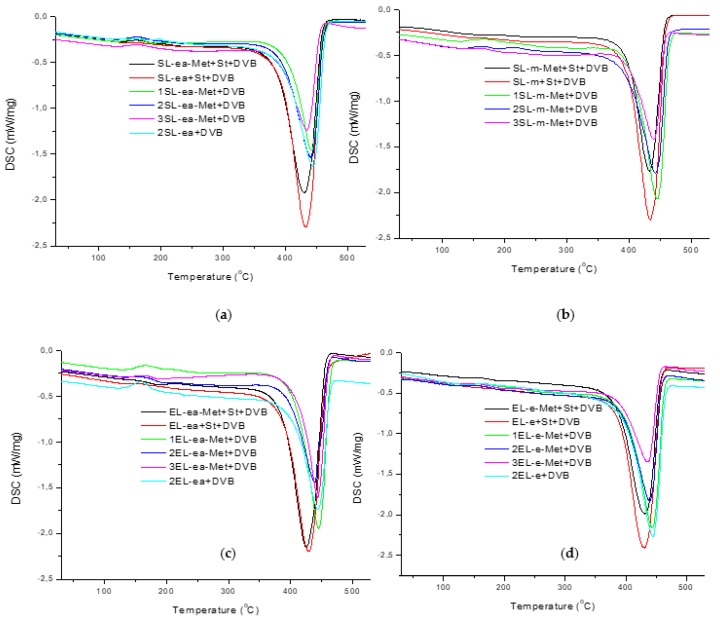
DSC curves of polymers containing (**a**) SL-ea, (**b**) SL-m, (**c**) EL-ea, (**d**) EL-e.

Thermal properties of the lignin-containing materials were studied by means of differential scanning calorimetry (DSC). The DSC curves are presented in Figure 3 and the maximum decomposition values (T_d_) and enthalpy of decomposition (ΔH_d_) are given in Table 3. In general, the synthesized materials are characterized by good thermal resistance. The DSC analysis showed that the thermal behavior of the materials obtained is similar, with a well-shaped calorimetric profile containing a single endothermic peak. A small exothermic effect at around 170 °C associated with the post-crosslinking process was also observed, particularly for the DVB-based materials containing SL-ea and EL-ea lignin, even though it was invisible for those with EL-e lignin, which can suggest that microspheres with EL-e lignin had the highest degree of crosslinking. The maximum decomposition temperatures for pure DVB and St-DVB microspheres are 447.2 °C and 426.9°, correspondingly, as shown in our previous paper [50]. The use of methacrylated lignin resulted in a slight deterioration of the thermal properties of the materials and, with increasing lignin content, the maximum decomposition temperature decreased. These values differed slightly (±5–6 °C) among microspheres with different lignin types and different fractions. Among the materials with the highest content of lignin (3 g), the highest maximum decomposition temperature was observed for the microspheres containing EL-ea-Met lignin. The use of lignin in St-DVB microspheres resulted in the improvement of thermal stability, especially when samples with molecular weight above 1000 g/mol were used [50]. The enthalpy of decomposition (ΔH_d_) depends on the amount of lignin in the materials and decreases with increasing lignin content.

**Figure 4:**
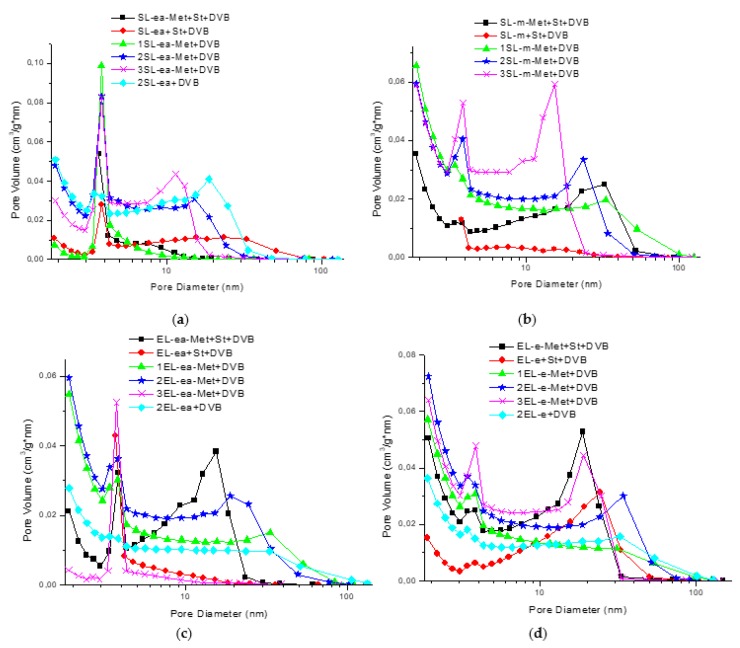
Pore size distribution curves of polymers containing (**a**) SL-ea, (**b**) SL-m, (**c**) EL-ea, and (**d**) EL-e.

The surface area and porosity results are presented in Table 4. The addition of a lignin component slightly decreased the porosity of the DVB-containing or St-DVB-containing microspheres [50]. On the other hand, lignin additive enables the incorporation of various functionalities, which is essential for sorption processes. Among the materials containing DVB and modified lignin, the higher molecular weight of initial lignin fraction resulted in more developed porosity of corresponding microspheres within a series (SL-ea vs. SL-m and EL-ea vs. EL-e). The highest S_BET_ values were obtained for the materials with 2 g of modified lignin irrespective of lignin origin. Polymer microspheres with EL-e-Met lignin had the highest specific surface area and the largest pore volume among all studied materials, which can be related to a formation of a highly cross-linked network. EL-e lignin fraction had a less condensed macromolecular structure with a high content of phenolic and aliphatic hydroxyl groups in phenylpropanoid units, as shown in Table 1. The higher percentage of these groups as compared to softwood lignin fraction could react with methacryloyl chloride [29], and this could give a more homogeneous distribution of methacrylic groups in the lignin macromolecules and a more crosslinked network in the final microspheres. The inclusion of styrene, however, led to a decrease in porosity. Figure 4 shows the pore size distribution of the materials, which reveals more information about the porosity. All the materials are mesoporous. The pore size distributions are bimodal with a narrow peak with a maximum at 3 nm and a broader peak with a maximum at 12–50 nm. The presence of modified lignin in the structure of microspheres results in a shift of the second peak toward a lower value suggesting the creation of narrower, deeper, and more uniform pores in the material. The larger the amount of modified lignin used, the narrower and deeper the pores are in the materials. The fractionated lignin results in a greater porosity of the synthesized materials than in the microspheres where commercially available and low molecular weight lignin was applied [50].

The results of swelling studies of the polymer materials are presented in Table 5. The lowest swellability coefficients were obtained for the polymers with EL-e lignin, which can be related to its crosslinking ability. Ethyl acetate fractions of lignin (SL-ea and EL-ea) had a higher swellability coefficient than EL-e and SL-m. Except for the materials with EL-e lignin, the greatest swelling tendency was observed in the materials with styrene and unmodified lignin. An increase in lignin content usually led to an increased swelling. In general, crosslinked materials have a low tendency to swell, but the presence of functional groups from lignin caused networks to interact with the solvents. None of the microspheres swelled in distilled water. The highest swelling coefficients were obtained in acetone and chloroform.

**Figure 5:**
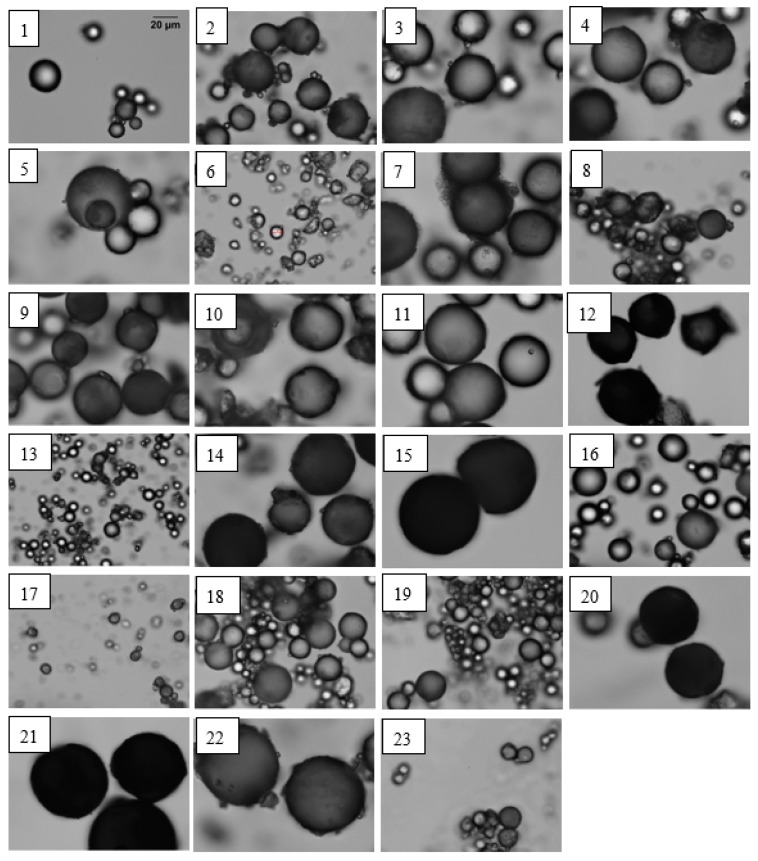
Photomicrographs of the microspheres. 1-SL-ea-Met + St + DVB, 2-SL-ea + St + DVB, 3-1SL-ea-Met + DVB, 4-2SL-ea-Met + DVB, 5-3SL-ea-Met + DVB, 6-SL-ea + DVB, 7-SL-m-Met + St + DVB, 8-SL-m + St + DVB, 9-1SL-m-Met + DVB, 10-2SL-m-Met + DVB, 11-3SL-m-Met + DVB, 12-EL-ea-Met + St + DVB, 13-EL-ea + St + DVB, 14-1EL-ea-Met + DVB, 15-2EL-ea-Met + DVB, 16-3EL-ea-Met + DVB, 17-EL-ea + DVB, 18-EL-e-Met + St + DVB, 19-EL-e + St + DVB, 20-1EL-e-Met + DVB, 21-2EL-e-Met + DVB, 22-3EL-e-Met + DVB, 23-EL-e + DVB. The scale bar (see sample 1) is the same for all images.

The spherical shape of the obtained materials was confirmed by photomicrographs, as shown in Figure 5. All the samples contain microspheres of different sizes. Materials with unmodified lignin had much smaller diameters (9–21 µm) than those with modified lignin (35–63 µm). Materials with modified lignin and DVB had the largest diameters in the range of 45–63 µm, and the microspheres with modified lignin from hardwood (EL-ea-Met and EL-e-Met) had larger diameters (49–63 µm) than those with modified lignin from softwood (SL-ea-Met and SL-m-Met). The diameters ranged from 45 to 52 µm. All the synthesized microspheres had a tendency to agglomerate. The most homogeneous size distribution was obtained for the materials using EL-e-Met lignin and DVB.

## 4. Conclusions

Two types of kraft lignin, including softwood (SL) and hardwood (EL) fractionated in common industrial solvents, were successfully modified with methacryloyl chloride. The modified and unmodified lignins were then used for the synthesis of polymeric microspheres with St and/or DVB. The structural characteristics of the lignin fractions, such as degree of condensation, number and type of various hydroxyl groups, and molecular weight, influenced the modification process and the properties of the materials. Thermal properties of materials were studied by DSC and the materials were found to have a high thermal resistance. The incorporation of methacrylated lignin into the microspheres resulted in the greatest specific surface area and porosity. The largest specific surface area and the largest total pore volumes were found in the materials with SL-m and EL-e fractions, which had a higher molecular weight than the corresponding ethyl acetate fractions SL-ea and EL-ea. SL-m and EL-e fractions had a higher content of aliphatic hydroxyl groups than the ethyl acetate fractions. These groups can also react with methacryloyl chloride, and, thus, can give a more homogeneous distribution of methacrylic groups in the lignin macromolecules and, therefore, better characteristics of the final products. The microspheres swelled most in acetone and chloroform. They did not swell at all in distilled water. Materials with EL-e had the lowest swellability coefficients, which may be related to their high degree of crosslinking. The most homogeneous size distribution of microspheres was obtained with EL-e-Met lignin and DVB.

## Figures and Tables

**Table 1:** Experimental parameters of the syntheses.

Polymer	Monomers (g)		
	L ^1^	L-Met ^2^	St ^3^
SL-ea-Met+St+DVB	0	2	4
SL-ea +St+DVB	2	0	4
1SL-ea-Met+DVB	0	1	0
2SL-ea-Met+DVB	0	2	0
3SL-ea-Met+DVB	0	3	0
2SL-ea+DVB	2	0	0
SL-m-Met+St+DVB	0	2	4
SL-m +St+DVB	2	0	4
1SL-m-Met+DVB	0	1	0
2SL-m-Met+DVB	0	2	0
3SL-m-Met+DVB	0	3	0
EL-ea-Met+St+DVB	0	2	4
EL-ea +St+DVB	2	0	4
1EL-ea-Met+DVB	0	1	0
2EL-ea-Met+DVB	0	2	0
3EL-ea-Met+DVB	0	3	0
2EL-ea+DVB	2	0	0
EL-e-Met+St+DVB	0	2	4
EL-e +St+DVB	2	0	4
1EL-e-Met+DVB	0	1	4
2EL-e-Met+DVB	0	2	0
3EL-e-Met+DVB	0	3	0
2EL-e+DVB	2	0	0

^1^ unmodified lignin, ^2^ modified lignin, and ^3^ styrene.

**Table 2:** Molecular weight characteristics and content of functional groups in lignin solvent fractions.

Lignin Fraction	Mn ^1^ (g/mol)	Mw ^2^ (g/mol)	Đ ^3^	Aliphatic-OH (mmol/g)	Carboxyl-OH (mmol/g)	Phenolic-OH (mmol/g)		
						Condensed G ^4^	Non-Condensed (G+S ^5^)	Total
Spruce-ethyl acetate (SL-ea)	720	1160	1.6	0.7	0.7	1.8	3.2	5.0
Spruce-methanol (SL-m)	1400	2900	2.1	1.8	0.4	2.0	2.5	4.5
Eucalyptus-ethyl acetate (EL-ea)	630	940	1.5	0.6	0.3	0.8	4.1	4.9
Eucalyptus-ethanol (EL-e)	870	1420	1.6	1.4	0.4	0.9	3.5	4.4

^1^ Number average molecular weight, ^2^ weight average molecular weight, ^3^ and polydispersity index. ^4^ Guaiacyl, ^5^ syringyl.

**Table 3:** DSC data.

Polymer	Td ^1^ (°C)	ΔHd ^2^ (J/g)
SL-ea-Met + St + DVB	430.2	539.8
SL-ea + St + DVB	432.7	595.1
1SL-ea-Met + DVB	441.6	321.0
2SL-ea-Met + DVB	440.6	378.0
3SL-ea-Met + DVB	434.3	296.8
2SL-ea + DVB	444.1	428.3
SL-m-Met + St + DVB	432.2	568.9
SL-m + St + DVB	434.3	529.9
1SL-m-Met + DVB	444.4	409.3
2SL-m-Met + DVB	442.0	420.5
3SL-m-Met + DVB	439.5	251
EL-ea-Met + St + DVB	425.3	558.7
EL-ea + St + DVB	428.6	556.3
1EL-ea-Met + DVB	445.1	407.7
2EL-ea-Met + DVB	438.3	318.9
3EL-ea-Met + DVB	443.7	315.2
2EL-ea + DVB	444.2	326.7
EL-e-Met + St + DVB	431.5	505.1
EL-e + St + DVB	430.5	579.6
1EL-e-Met + DVB	443.6	446.9
2EL-e-Met + DVB	439.4	377.2
3EL-e-Met + DVB	435.2	281.6
2EL-e + DVB	444.7	479.0

^1^ Temperature of maximum decomposition, and ^2^ enthalpy of decomposition.

**Table 4:** Pore structure parameters of the polymers.

Polymer	S_BET_^1^ (m^2^/g)	V_TOT_^2^ (cm^3^/g)	D_A_^3^ (nm)
SL-ea-Met + St + DVB	51	0.104	8.2
SL-ea + St + DVB	139	0.597	17.1
1SL-ea-Met + DVB	103	0.126	4.9
2SL-ea-Met + DVB	396	0.700	7.1
3SL-ea-Met + DVB	314	0.542	6.9
2SL-ea + DVB	416	0.996	9.6
SL-m-Met + St + DVB	291	0.983	13.5
SL-m + St + DVB	23	0.077	13.2
1SL-m-Met + DVB	474	1.405	11.9
2SL-m-Met + DVB	442	0.969	8.8
3SL-m-Met + DVB	462	0.803	6.9
EL-ea-Met + St + DVB	229	0.517	9.0
EL-ea + St + DVB	30	0.080	10.6
1EL-ea-Met + DVB	384	0.937	9.7
2EL-ea-Met + DVB	434	0.953	8.8
3EL-ea-Met + DVB	62	0.083	5.4
2EL-ea + DVB	212	0.802	15.1
EL-e-Met + St + DVB	410	0.938	9.1
EL-e + St + DVB	195	0.785	16.1
1EL-e-Met + DVB	394	0.996	10.1
2EL-e-Met + DVB	506	1.342	10.6
3EL-e-Met + DVB	483	0.981	8.1
2EL-e + DVB	299	1.135	15.2

^1^ specific surface area, ^2^ total pore volume, ^3^ and average pore diameter.

**Table 5:** Swelling studies.

Polymer	Swellability Coefficient, B (%)						
	Acetone	THF ^1^	Chloroform	ACN ^2^	Methanol	Toluene	Aqua dest.
SL-ea-Met + St + DVB	122	122	122	122	100	100	0
SL-ea + St + DVB	100	30	91	67	58	100	0
1SL-ea-Met + DVB	113	78	75	63	63	63	0
2SL-ea-Met + DVB	67	46	85	82	64	75	0
3SL-ea-Met + DVB	58	83	45	55	80	45	0
2SL-ea + DVB	73	47	67	67	60	83	0
SL-m-Met + St + DVB	0	0	0	10	0	0	0
SL-m + St + DVB	100	60	120	100	80	209	0
1SL-m-Met + DVB	8	8	8	15	0	0	0
2SL-m-Met + DVB	0	0	0	0	0	11	0
3SL-m-Met + DVB	22	10	11	11	11	10	0
EL-ea-Met + St + DVB	23	25	55	36	27	55	0
EL-ea + St + DVB	109	120	136	91	118	127	0
1EL-ea-Met + DVB	7	13	7	7	7	22	0
2EL-ea-Met + DVB	20	27	6	8	15	7	0
3EL-ea-Met + DVB	40	40	80	70	40	70	0
2EL-ea + DVB	0	6	0	0	0	6	0
EL-e-Met + St + DVB	22	10	11	0	0	0	0
EL-e + St + DVB	0	9	0	0	0	0	0
1EL-e-Met + DVB	6	0	0	10	0	0	0
2EL-e-Met + DVB	22	0	0	11	0	0	0
3EL-e-Met + DVB	25	0	13	13	13	14	0
2EL-e + DVB	0	0	0	0	0	0	0

^1^ Tetrahydrofuran, and ^2^ acetronitrile.
